# Simplifying software compliance: AI technologies in drafting technical documentation for the AI Act

**DOI:** 10.1007/s10664-025-10645-x

**Published:** 2025-04-02

**Authors:** Francesco Sovrano, Emmie Hine, Stefano Anzolut, Alberto Bacchelli

**Affiliations:** 1 ETH Zurich, Collegium Helveticum, Zurich, Switzerland; 2 University of Zurich, Zurich, Switzerland; 3 Yale Digital Ethics Center, New Haven, CT USA; 4 University of Bologna, Bologna, Italy; 5 KU Leuven, Leuven, Belgium

**Keywords:** AI Act, Legal compliance, Technical documentation, Applied computing in law, Software documentation, 68N30, 68T07, 91C99

## Abstract

The European AI Act has introduced specific technical documentation requirements for AI systems. Compliance with them is challenging due to the need for advanced knowledge of both legal and technical aspects, which is rare among software developers and legal professionals. Consequently, small and medium-sized enterprises may face high costs in meeting these requirements. In this study, we explore how contemporary AI technologies, including ChatGPT and an existing compliance tool (DoXpert), can aid software developers in creating technical documentation that complies with the AI Act. We specifically demonstrate how these AI tools can identify gaps in existing documentation according to the provisions of the AI Act. Using open-source high-risk AI systems as case studies, we collaborated with legal experts to evaluate how closely tool-generated assessments align with expert opinions. Findings show partial alignment, important issues with ChatGPT (3.5 and 4), and a moderate (and statistically significant) correlation between DoXpert and expert judgments, according to the Rank Biserial Correlation analysis. Nonetheless, these findings underscore the potential of AI to combine with human analysis and alleviate the compliance burden, supporting the broader goal of fostering responsible and transparent AI development under emerging regulatory frameworks.

## Introduction

The artificial intelligence (AI) Act, adopted by the European Parliament on 13 March 2024, is going to regulate AI across the European Union (EU), impacting development and software engineering processes (Commission 2021; Sovrano and Masetti 2022). Similarly to the General Data Protection Regulation (GDPR) of the European Parliament and Council of the European Union (2016), the AI Act will affect not only EU-based industries but also non-EU companies doing business in EU.

The AI Act underscores the importance of AI-specific concerns, particularly transparency, fairness, and risk management (Sovrano et al. 2021, 2022). As a result, many AI practitioners may have to reassess and adapt their standard software engineering practices, thus posing new challenges for existing organizations and companies (Jha and Leahy 2024; Musch et al. 2023; Svantesson 2022). OpenAI’s CEO, Sam Altman, voiced concerns about the technical constraints of the AI Act, highlighting potential impacts on the company’s ability to comply and operate within the EU.^1^

Even though the European Commission (2022) aims to foster innovation, particularly among start-ups and other small and medium-sized enterprises (SMEs), concerns exist that the AI Act might hinder European innovation and competitiveness, owing to compliance costs and the complexity of understanding the AI Act’s stipulations (Digital Europe 2023).

The legislation mandates several requirements for (high-risk) AI systems, necessitating tailored adjustments in software documentation (Article 11 of the AI Act; Annex IV). Among other things, these requirements encompass design choices, training data, algorithmic logic, performance metrics, potential biases, and risk mitigation strategies, all of which must be documented into technical software documentation.

While typical AI technical documentation already includes details on system design, architecture, and testing procedures (Königstorfer and Thalmann 2022; Garousi et al. 2015), the AI Act introduces more rigorous standards. Adhering to these standards will necessitate modifications to existing documentation practices in software engineering and require advanced knowledge of both legal and technical aspects. This work focuses precisely on addressing this new interdisciplinary challenge to streamline the production and validation of software documentation compliant with the AI Act.

Indeed, SMEs and independent AI developers must create AI software and technical documentation that meet the AI Act’s requirements. However, the drafting and review of technical documentation for AI Act compliance by individuals without legal expertise are challenging. Likewise, legal experts may find it daunting to navigate highly technical software documentation. This knowledge gap can lead to significant legal fees and time costs for all parties involved.

Consequently, any tool that can expedite the assessment, drafting, and maintenance of technical software documentation required by the AI Act will alleviate the financial burden on SMEs and independent AI developers, both within the EU and globally.

In this paper, we investigate the extent to which existing AI technology can aid developers in creating high-quality technical documentation under the AI Act. Additionally, we examine the limitations of these technologies and the areas where human intervention is necessary.

**Fig. 1:**
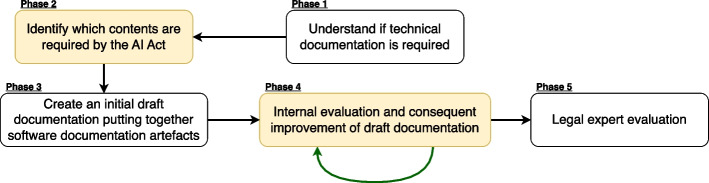
Diagram illustrating the methodology for drafting AI Act-compliant technical documentation. The process begins with determining the need for such documentation, followed by identifying essential content. An initial draft is compiled from existing software documentation artifacts and then internally evaluated for enhancements using automated tools. After the draft is finalized, it is reviewed by a legal expert for further refinement. 
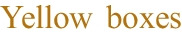
 highlight steps in the drafting process we contributed to, while the 
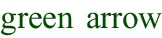
 indicates our main methodological contribution: the automation of the internal review process

We modelled the process of creating such documentation as comprising five main phases, fleshed out in Section 3 and shown in Fig. 1.

To implement this process, we first identified the documentation requirements of the AI Act and formatted them into a questionnaire. Next, we created two initial drafts of technical documentation based on open-source high-risk AI systems (*i.e.*, for credit assessment and medical expense estimation) from IBM Research’s AIF360 (IBM 2023b, c) and AIX360 (IBM 2023a, d) libraries. We then adapted existing legal compliance checking pipelines (Sovrano et al. 2024a) to align the original software documentation of these high-risk systems with the AI Act’s requirements. This is performed to illustrate a plausible scenario wherein non-compliant software documentation (*e.g.*, a draft or outdated documentation) needs to be converted into a format compliant with the AI Act.

To determine whether these strategies can help producing high-quality technical documentation, we conducted a legal evaluation involving three independent legal experts. Our findings revealed that pipelines relying solely on ChatGPT tend to perform poorly, especially with longer documents. Our examinations of ChatGPT revealed similar limitations to those highlighted by Dahl et al. (2024) and Sovrano et al. (2024a), such as constraints related to input size and memory, as well as a tendency to generate hallucinated information, particularly with simple prompts (Alkaissi and McFarlane 2023; Azamfirei et al. 2023).

Our main contributions include:

- A study of how software engineering practices will be impacted by the AI Act.
- A methodology to enhance the review process for technical software documentation under the AI Act by identifying missing information in draft documentation (see Fig. 1).
- An evaluation on how ChatGPT and current AI technology can aid developers in producing high-quality technical documentation under the AI Act.
- The source code for the automated assessment tools and the data we reference in this paper (Sovrano 2025).

Finally, as the AI Act will be fully in effect around 2026 and demands a novel type of technical documentation, no prototypical examples of such documentation are already available online^2^. Therefore, we also contribute with prototypical examples of technical documentation for the AI Act and explanations on how to create them.

## Background and Related Work

In this section, we present the AI Act, its regulatory impact, the importance of transparency and fairness in this context, and some tools for automated compliance assessment. We also compare our study with other research on the AI Act and on compliance assessment.

### The AI Act

On April 21, 2021, the EU Commission introduced the AI Act (Commission 2021), the first comprehensive law aimed at regulating AI technologies, approved by the EU Parliament on 13 March 2024 and voted by the EU Council on 21 May 2024. The Act takes a broad view of AI, including machine learning algorithms, expert systems, and traditional statistical models.

According to Sovrano et al. (2022), the proposed AI Act aims to ensure transparency, lawfulness, and fairness in AI systems, emphasizing quality from inception through the entire lifecycle. Pfeiffer et al. (2023) instead highlights that the AI Act adopts a risk-based approach, focusing primarily on non-discrimination and preventing harmful practices, rather than explicitly addressing the broader concept of fairness. The Act links to the EU Charter of Fundamental Rights^3^, indirectly invoking fairness while directly tackling non-discrimination in its regulatory framework.

The AI Act takes a tiered, risk-based approach to AI risk assessment, defining three risk layers: systems that pose an unacceptable risk, like social scoring, are prohibited (Art. 5; AI Act); high-risk applications, such as CV-scanning tools for job applicants, must meet specific legal requirements (Art. 6; AI Act); applications not explicitly banned or deemed high-risk are generally unregulated, though foundation models and AI systems designed for human interaction must comply with transparency requirements (Arts. 28b & 52; AI Act). High-risk applications, whether listed in Annex III or qualifying based on harmonization legislation, are required to produce extensive technical documentation before entering the market or being deployed (Art. 11 & Annex IV; AI Act). This documentation must also be regularly updated.

The primary goal of the AI Act is to manage risks while promoting trustworthy AI and innovation. To bolster this legislative initiative, the EU Commission has established a symbiotic relationship between the AI Act and *harmonized standards* (*i.e.*, technical standards developed by European Standardization Organizations to provide uniform guidelines for implementing EU directives).

Compliance with these officially adopted harmonized standards creates a “presumption of compliance” with the AI Act’s requirements (Art. 40; AI Act). Once standards are ratified and published, industries can adopt them, which helps to unify regulations, clarify compliance procedures for companies, and facilitate the safe deployment of AI technologies across the EU. As of June 2024, there are no harmonized standards for the kind of technical documentation defined in Annex IV of the AI Act.

Since 2021, several methods for compliance with the AI Act have been developed. An early initiative by Floridi et al. (2022), known as capAI, introduces a procedure for conformity assessments based on the AI Act. However, it does not have a guide specifically for creating or assessing technical software documentation as per Annex IV.

Shortly after, Mökander et al. (2022) argued that the AI Act is “a proposal to establish a Europe-wide ecosystem for conducting AI auditing”. There is an extant body of work on AI auditing, including research on large language models (LLMs) (Mökander et al. 2023), explainability (Vilone et al. 2024; Sovrano et al. 2024b), supplier’s declarations of conformity (Arnold et al. 2019), continuous auditing (Minkkinen et al. 2022), and, most significantly, ethics-based auditing (Mökander et al. 2021; Mökander and Floridi 2021; Schöppl et al. 2022; Mökander and Floridi 2023). Differently from all of these works, we specifically focus on the AI Act’s technical documentation (*i.e.*, Annex IV), with the objective of streamlining the documentation drafting process thus reducing its associated costs.

Although it is challenging to provide an exact estimation of the legal consultation costs involved without a detailed case-by-case analysis, we can anticipate that the expenses for SMEs are significant. This inference stems from the current shortage of legal experts specialized in the AI Act, as noted by Koh et al. (2024). In EU countries like Austria and Germany, standard legal rates are already substantial, with fees typically ranging from € 20 to € 150 per hour and € 65 to € 125 per hour, respectively.^4^ These rates serve as a lower bound; actual costs often exceed these figures, influenced by factors such as the law firm’s prestige, the seniority of the legal expert, market demand, and the risks of legal consultations.

### Automated Compliance Assessment Tools

Our research builds upon the investigation conducted by Sovrano et al. (2024a) on automated tools for checking compliance of software documentation with the EU Platform to Business (P2B) Regulation. The P2B Regulation requires online platforms, such as Google and Amazon, to provide documentation that is accessible, understandable, and detailed about their ranking mechanisms and how these affect sellers, including business and corporate website users. Typically, this type of software documentation is less technical and shorter than that required by the AI Act, as it targets laypeople.

In this paper, we aim to broaden the scope of Sovrano et al. (2024a)’s analysis to include more complex types of software documentation, to assess whether the proposed tools are effective in the context of the AI Act. Hence, we employed the same tools for automated compliance checking as those used in Sovrano et al. (2024a)’s study (*i.e.*, ChatGPT and an answer retrieval approach) and investigate how LLMs can assess the quality of technical explanations and identify areas where information is missing. Our work further explores how AI can enhance the documentation creation process for software engineers and demonstrates the broader applicability of the technology in legal compliance verification tasks.

ChatGPT, a state-of-the-art LLM capable of processing and generating text, has been deployed for automated assessment tasks in diverse contexts such as evaluating student answers (Li et al. 2023) and analyzing data (Ma et al. 2023). Beyond AI, various organizations have developed manual tools for legislative compliance, particularly targeting the GDPR (Agarwal et al. 2018; Zhang et al. 2024). Our work uniquely focuses on the technical documentation requirements of the AI Act.

An emerging solution that could further support these documentation requirements is the *AI Bill of Materials* (AI BOM) (Santos and Radanliev 2023; Camp 2023). Much like a traditional Bill of Materials in manufacturing, an AI BOM provides a detailed inventory of all components in an AI system, including the model details, architecture, usage, and training data. Notably, Annex IV of the AI Act requires in part some kind of BOM (see TD.1 and TD.2, Table 1). Indeed, by detailing the building blocks of an AI system, an AI BOM can help stakeholders understand how the system is constructed and maintained, thereby supporting more robust and auditable compliance checking. While we do not delve into AI BOMs in this paper, they represent a complementary research avenue that could further strengthen automated compliance solutions by offering a standardized, transparent view of the components that drive AI systems.

As outlined before, our research builds upon the tools introduced by Sovrano et al. (2024a).

Below, we provide explanations of these tools to enhance clarity and facilitate a better understanding of how they were adapted for our current study on the AI Act.

Specifically, these tools are:

- a standalone ChatGPT-based pipeline;
- an answer retrieval pipeline augmented with ChatGPT.

Both of these pipelines process a list of questions (*e.g.*, a checklist) and a text (software documentation) to generate compliance scores. The output for each question includes one or more scores; specifically, the second pipeline produces three scores per question, as detailed below.

#### Standalone ChatGPT-Based Pipeline

The standalone ChatGPT-based pipeline processes software documentation by breaking it down into text chunks that adhere to the token limits of ChatGPT. Each chunk is then individually assessed against the questions, receiving a score from 1 (non-compliant) to 5 (fully compliant). The algorithm aggregates the scores by taking the maximum score for each checklist item.

In the case of GPT-4, these chunks consist of 8192 tokens, while for GPT-3.5 (version 0613, from June 13th 2023), the limit is 16385 tokens. In our case studies, the documents processed by ChatGPT-3.5 do not exceed 16385 tokens, so each document is contained within a single chunk. However, even when large context windows are available, recent research suggests that simply enlarging the context window is not a cure-all for the challenges LLMs face in retaining and effectively using relevant information in lengthy inputs (Levy et al. 2024).

To produce the score and process the documentation chunks, the following prompt template is used:

*As a machine learning engineer and legal expert, your task is to assess the compliance of this technical documentation about an AI system based on the following question. Conduct an intermediate compliance assessment, focusing on both the technical and legal requirements.*

*Your assessment should start with a numerical score from 1 to 5, where 1 indicates the question is not answered at all and 5 indicates it’s perfectly answered. Following the score, provide a brief explanation highlighting the strengths or weaknesses in addressing the question. Consider the completeness, clarity, and legal implications in your explanation.*

*For example, your assessment might look like: ’Score: 3. Explanation: The question was only partially answered. While the technical aspects are covered, it lacks legal disclosures.’*

*Question:* {*question*}

*Technical documentation:* {*chunk*}

The prompt template was defined through an iterative, experimentation-driven process. Early versions asked only for a concise compliance assessment, but they frequently produced incomplete or inconsistent outputs that were difficult to analyse via pattern matching. The final prompt was thus engineered to provide an example of the expected output. This prompting strategy is called *in-context learning* (Marvin et al. 2024) or few-shot learning. We additionally leveraged techniques recommended by OpenAI:^5^ specifying the desired context and outcome, offering detailed, step-by-step instructions (*i.e.*, “first give a score, then explain it”), and providing ChatGPT with a specific persona^6^ (machine learning engineer and legal expert).

#### DoXpert: Retrieval-Augmented ChatGPT

Although many modern LLMs, including ChatGPT, are capable of processing extensive inputs, they often struggle to fully leverage information within a lengthy context, a challenge referred to as the *lost-in-the-middle* or *lost-in-the-end* issue (Liu et al. 2024; Sovrano et al. 2025; An et al. 2024; Xu et al. 2024; Levy et al. 2024).

Research by Xu et al. (2024) has shown that retrieval methods can significantly enhance the performance of LLMs, regardless of their extended context window sizes. Building on this, Sovrano et al. (2024a) introduced a novel strategy which we called DoXpert. This approach uses advanced prompt engineering and answer retrieval technology to effectively summarize lengthy documents, similar to what a retrieval-augmented generation (RAG) system does. It addresses the hallucination issue associated with the lost-in-the-middle and lost-in-the-end problems by employing ChatGPT solely for paraphrasing the provided information, rather than generating summary assessments.

Similarly to the standalone ChatGPT-based pipeline, also the DoXpert tool takes as input a set of questions and a text to evaluate. Differently though, it does not take as input only a checklist but also an open-ended questionnaire. Indeed, a checklist is framed for binary responses, but answer retrieval tools (such as DoXpert) are usually more suited for open-ended queries, being designed to retrieve paragraph-length responses. Consequently, for each checklist question, DoXpert needs an equivalent question formulated in an open-ended format.

In the DoXpert pipeline, the process begins by dividing the input document into paragraphs. These paragraphs are then transformed into a vectorial form using a sentence embedding neural network. Following this, an answer retriever processes each open-ended question individually, converting it into a vectorial representation as well. The vectors for the open-ended questions are then matched with the vectors of the paragraphs, and the top-k paragraphs that show the highest similarity (*i.e.*, pertinence) are selected.

Each selected paragraph is assigned a *pertinence score* ranging from 0 to 1, where scores closer to 1 indicate greater relevance to the question. This score represents the cosine similarity between the vector of the question and the vector of the paragraph.

Then, ChatGPT 4^7^ is used to sift through the retrieved answers and amalgamate them into a comprehensive explanation. Specifically, ChatGPT’s role is to identify and filter out incorrect responses, aggregating and paraphrasing the correct ones into binary answers fitting the checklist. Indeed, the checklist format is preferred over an open-ended questionnaire because it is usually simpler to process for a human (*e.g.*, a legal expert) (Adams 2015) and allows for comparable quantitative measurements (which we will need for answering our second research question; cf. Section 3.2).

For doing so, the used prompt is:

*Output a comprehensive answer based only and exclusively on the information within the paragraphs below (if any can be used to answer), which were extracted from the documentation to be assessed. If no paragraph can answer the question, then output only “No, I cannot answer”. Otherwise, the comprehensive answer must contain citations to the source paragraphs,* *e.g.*, *blablabla (paragraphs 1 and 2), blabla (paragraph 0). It should also start with “Yes” if the answer is positive, “No” if the answer is negative, or “N/A” if the answer is not available.*

*Question:* {*question*}

*Paragraphs:* {*contents*}

This process is akin to what a standard RAG system does. Indeed, in RAG-based methods, the LLM is supplied with a retrieval step (*i.e.*, the “chunking” and feeding back relevant paragraphs) and then asked to generate an answer that incorporates that retrieved context. Unlike RAG and the standalone ChatGPT-based pipeline previously mentioned, DoXpert generates three scores for each question. The first score is a boolean value, telling whether a given question is answered in the input text, as instructed by the prompt above. The second and third scores, known as the DoX score and the explanatory relevance score, assess the quality of the answers provided. Previous research has shown that these scores may correlate well with assessments made by legal experts (Sovrano et al. 2024a).

In particular, once the filtration and aggregation process by ChatGPT is complete, DoXpert applies the DoX metric to assess the explanatory depth of these refined answers, thus serving as a measure of how well the retrieved and refined content addresses the compliance checklist’s criteria.

The DoX metric, introduced in the field of explainable artificial intelligence (XAI) by Sovrano and Vitali (2023), is designed to assess the clarity and depth of text explanations. It is particularly useful for evaluating whether documentation offers clear and comprehensive explanations. For instance, consider a scenario where an AI developer is asked, “How does your system mitigate biases?” A response such as “We use a bias-correction algorithm” might be accurate but lacks depth. According to the principles of Ordinary Language Philosophy (Achinstein 2010; Sovrano 2023) , a thorough explanation would be: “We employ a bias-correction algorithm that cross-references training data with multiple diverse datasets, ensuring the AI’s predictions remain fair across different demographic groups.” DoX awards a higher score to the latter explanation for its greater explanatory depth, penalizing superficial responses.

While other explainability metrics exist, they generally require human evaluations—*e.g.*, usability (Dieber and Kirrane 2022; Sovrano and Vitali 2021)—or are limited to logical statements, not natural language—*e.g.*, number of rules (Rosenfeld 2021), monotonicity (Nguyen and Martínez 2020), *etc.* To our knowledge, and as supported by literature reviews (Sovrano et al. 2021; Sovrano and Vitali 2022), DoX is the only metric available capable of assessing any natural language explanation without the involvement of a pool of subjects, *i.e.*, a user study.

The other metric instead, the explanatory relevance score, is calculated using the formula:

$$\begin{aligned} \text {Explanatory Relevance Score} = \text {DoX} \times \text {Max(Pertinence Score)} \end{aligned}$$

Here, *Max(Pertinence Score)* is essentially the highest pertinence score among the retrieved correct answers for an open-ended question. The explanatory relevance score thus serves as an aggregate metric that captures both the depth of explanations and the specificity of content in terms of compliance with the regulation embodied in the considered questionnaires.

## Methodology

As anticipated in Section 1, see in Fig. 1, creating technical documentation that conforms to the AI Act involves five main phases:

- **Phase 1**: Understand when technical documentation is required.
- **Phase 2**: Identify which contents are required by the AI Act.
- **Phase 3**: Create an initial draft documentation.
- **Phase 4**: Internal review and improvement of the draft documentation.
- **Phase 5**: Legal expert evaluation.

Initially, it is essential to determine whether technical documentation is necessary for the AI system in question (**Phase 1**). As previously mentioned, only AI systems categorized as high risk (see Section 2 for more details) must have such documentation. Next, one must identify the core contents mandated by the AI Act for technical documentation and assess their implications on the development, deployment, and maintenance of the AI system (**Phase 2**).

To address **Phase 2**, we developed a systematic approach by first conducting a thematic analysis (Creswell and Creswell 2018; Braun and Clarke 2021) of the contents of Annex IV of the AI Act, performed by the authors, which pinpointed specific provisions relevant to high-risk AI systems’ documentation. The version of the AI Act we considered is 2021/0106(COD), before the EU Parliament corrigenda of 13 March 2024. Subsequently, an inductive coding phase (Fereday and Muir-Cochrane 2006) performed by the authors (*i.e.*, law and software engineering researchers), facilitated the development of a comprehensive list of sections necessary for compliant technical documentation. This list covers information such as the system design, data origins, algorithmic transparency, and risk mitigation strategies. All the necessary details about **Phase 2** are provided in Section 4.

**Phase 3** involves assembling various software documentation artifacts to create an initial draft of the technical documentation. This process typically occurs during the development or prototyping of the AI system and includes detailing the methods used and the strategies implemented for training and evaluating the system. In **Phase 4**, the initial draft undergoes iterative reviews and refinements to develop a polished version of the documentation ready for the final step. This final phase (**Phase 5**) involves a thorough legal consultation, which may be costly, possibly followed by a new internal review process in case the documentation is not compliant yet.

We build on top of this five-step process, exploring the use of AI technology to decrease the frequency of legal consultations without compromising the number of internal reviews necessary to finalize the technical documentation. Our investigation is encapsulated in two main research questions.

### RQ1: Automating the Process of Reviewing and Refining Draft Documentation

Our first research question (**RQ1**) focuses on how to cut costs within the internal review process (**Phase 4**) by automating it, employing AI technology to identify missing information within draft documentation.
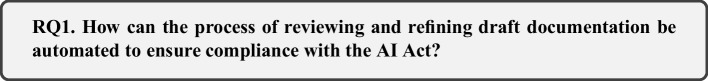


To understand how to automate the process of reviewing draft documentation, we first analysed the existing literature on compliance checking. We found out that evaluating the quality or compliance of a (technical) document can be conducted through various methods, including open-ended or closed-ended questionnaires. Open-ended questionnaires are usually better at capturing the intricacies of an assessment, typically catering to qualitative evaluations. However, their comparative analysis can be challenging due to the complexity and ambiguity inherent to natural language. Instead, closed-ended questionnaires, *e.g.*, checklists, can be translated into numerical scores, facilitating straightforward comparisons (Griffith et al. 1999).

Consequently, we decided to answer **RQ1** by designing both a closed-ended (*i.e.*, a checklist) and an open-ended questionnaire. To create these lists of questions from Annex IV of the AI Act, we conducted a systematic qualitative analysis of the text to pinpoint key compliance indicators. Employing an inductive coding method, similar to the one used for defining the organization and structure of compliant technical documentation, enabled us to derive questions directly from the text of Annex IV (cf. Section 5.1), all needing positive responses for a technical documentation to be compliant with the AI Act.

The process of creating the questionnaires involved paraphrasing into questions the contents of Annex IV and Articles 9, 13, and 14 of the AI Act. This resulted in a weakly sequential criteria of merit checklist (Scriven 2000). This simple extraction strategy allows for a seamless integration and modification of the questionnaires in response to changes in the AI Act.

With the compliance questions prepared, we were then able to identify automated strategies to assess software documentation against each question. Considering the nature of the task, the two most accessible technological tools identified were those used by Sovrano et al. (2024a): ChatGPT, and an answer retrieval solution. These assessment tools were crafted to automate the evaluation process of software documentation. They employ natural language processing techniques to analyse the text and generate detailed reports on the documentation’s adherence to regulatory standards based on the abovementioned closed-ended and open-ended questionnaires.

As of early 2024, these methods exemplify the two primary technological approaches currently leading the field in open-domain question answering: standalone LLMs and retrieval-augmented LLMs, respectively. While more complex and advanced strategies might be developed, we chose to focus exclusively on these compliance automation strategies to determine if even the simplest and most accessible methods are adequate for the task of assessing the compliance of software documentation under the AI Act. Analysing the impact of more advanced strategies is left as a future work.

For evaluating the automation of the internal review process (*i.e.*, the green arrow in Fig. 1), we considered the accuracy, precision, recall, and F1 scores of the assessment tools in identifying information missing in the initial draft documentation. Thus, in order to answer **RQ1**, we also had to fully implement the five-step process depicted in Fig. 1. This entailed not only identifying the main contents required by the AI Act (see Section 4), but also creating initial drafts of technical documentation by putting together existing software documentation artifacts, replicating a complete internal review process followed by a legal consultation.

To create the initial draft documentation (**Phase 3**), we conducted a thorough online search for well-documented open-source AI applications that incorporate XAI strategies (ensuring transparency) and strategies to mitigate bias (promoting fairness). These qualities align with the requirements set forth by the AI Act, as detailed in Section 2. Our search led us to IBM Research’s repositories, AIF360 (IBM 2023b, c) and AIX360 (IBM 2023a, d). These resources met all specified criteria: they are open-source, well-documented, and implement both fairness and transparency strategies in high-risk AI applications (**Phase 1**). They provide detailed technical explanations that are needed for crafting the kind of comprehensive initial draft documentation required for at least two high-risk systems: a credit approval system and a medical expenditure estimator (see Section 5.2 for more details). Since these AI tools are developed by IBM, a prominent technology firm, they serve as practical, albeit simplified, examples of AI applications used in the industry.

In drafting the initial draft documentation, we intentionally did not add any information mandated by Annex IV of the AI Act which could not be inferred from the documentation of AIF360 and AIX360, such as details on the risk management system and the EU Declaration of Conformity. For more details about this process. This deliberate omission enabled us to evaluate the effectiveness of the considered technologies in identifying and detecting missing information within realistic technical documentation.

The initial draft documentation was authored by the first and third authors of this paper, both experts in software engineering and computer science, but not in law. As they worked on each draft, they carefully tracked how each piece of information could address a specific requirement, with each requirement represented by a checklist question. If a piece of information could contribute even partially to a question according to at least one of the authors, it was considered a potential answer. This practice effectively resolved any disagreements between the two authors. It also avoided having to determine a variable minimum level of detail required to fully satisfy each question, a task that requires legal expertise. In fact, Phase 3 should not involve the participation of legal experts.

By structuring the documentation in this manner, we established a ground-truth dataset for assessing the performance of tools used during iterative draft reviews. Although this ground-truth is weaker than that obtained from legal experts, it is neither a primary contribution of the paper nor does it affect the results from the legal experts’ evaluations.

### RQ2: Quality of the Automated Assessment Tools

Instead, our second research question (**RQ2**) is about evaluating the usefulness of the AI-based tools considered (**Phase 5**). 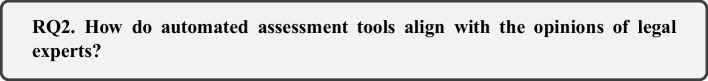


Specifically, for the evaluation of the draft documentation, we established one primary criterion: the degree of concurrence between the evaluations made by legal experts and the results produced by the assessment tools.

This metric is considered for assessing the final draft’s compliance and the tools’ capability in minimising discrepancies with the legal experts’ judgments. Indeed, fewer discrepancies imply fewer review rounds and less work for the legal experts to do, with a consequent reduction of the costs associated with legal consultations.

For the legal analysis, we required the expertise of at least three specialists in technology law and the AI Act. The decision to involve at least three specialists rests on the consideration that legal interpretations can vary among experts due to differences in perspective, experience, or emphasis on certain legal principles. To capture these disagreements, a Cohen’s Kappa analysis (Cohen 1960) is used.

Our search for potential candidates was challenging due to the limited number of experts specializing in the AI Act, as highlighted in recent literature (Koh et al. 2024). Our efforts led us to the web-page of the First University of St. Gallen Grand Challenge on the AI Act (Burri 2023), listing teams of qualified candidates for assessing AI Act compliance in technical documentation. Consequently, we reached out to the LegalAIzers team (Hine et al. 2023), winners of the Grand Challenge.

Although the Grand Challenge was not specifically about the technical documentation required by Annex IV of the AI Act, the aforementioned winning team has a provable track record of expertise in the AI Act, thus reasonably qualifying as very good candidates for the legal assessment we needed.

Two members from the LegalAIzers team agreed to be part of this study; one is a PhD candidate in a legal program with about 4 years of experience in the field, and the other is a senior lecturer holding a PhD in law (over 20 years of studies and experience). The former also became a co-author of this paper, after completing the evaluation, and also contributed to establishing the ground truth for the first evaluation criterion previously mentioned. For our third expert, we recruited another individual from the University of Bologna (Italy) with about 5 years of studies in law. This expert holds a master’s degree in law and has a strong background in European law and legal informatics.

Although the first two experts offer a deeply specialized understanding of the AI Act, including its technical and policy dimensions, the third expert’s background in legal informatics complements this focus with a broader view that integrates practical and technological considerations under the umbrella of European law. By incorporating all three experts, we capture a range of viewpoints: from senior academic expertise specifically targeting the AI Act, to a doctoral-level focus on legal doctrine and policy, to a more generalized legal expertise brought forth by the third expert. This diversity in expertise not only enriches the evaluation but also ensures that potential gaps, particularly at the intersection of law and emerging AI technologies, are better addressed.

These specialists were tasked to review our final documentation, *i.e.*, the result of the iterative automated review process, to understand to what extent it meets the legal standards of the AI Act.

## Guidelines for AI Act-Compliant Documentation

This section outlines recommendations for developing and organizing technical software documentation that complies with the AI Act. These guidelines are tailored for high-risk AI systems and serve two key purposes: first, to ensure that documentation meets regulatory standards; and second, to clarify how these regulations influence software engineering practices. Although the focus here is more narrative than empirical, this knowledge is crucial for practitioners seeking to create technical documentation in accordance with our methodology.

**Table 1:** *Technical Documentation Structure*

ID	Section	Main Contents
TD.1	General Description of the AI System	Developer info, installation instructions, intended purpose, description of market forms and hardware, version history and update requirements, interactions with other systems, product depictions, and user guidelines.
TD.2	Detailed Description of the AI System’s Elements and Development Process	Development methods, design choices, algorithms, trade-offs, architecture, computational and data requirements, human oversight, and validation metrics.
TD.3	Monitoring, Functioning, and Control of the AI System	Capabilities, limitations, unintended outcomes, risks, and input data requirements.
TD.4	Risk Management System	Continuous process aligning with Article 9 of the AI Act. Includes risk identification, evaluation, and management measures.
TD.5	Lifecycle Changes	Chronological catalog of changes.
TD.6	Harmonised Standards and Compliance	Enumerated standards or alternative approaches for compliance.
TD.7	EU Declaration of Conformity	As described in Annex V of the AI Act.
TD.8	Post-Market Monitoring	Periodic reassessment plan for post-deployment performance metrics.

Sections composing the technical documentation required by the AI Act, version 2021/0106(COD). **N.B.** As explained at the end of Section 4.1 of this paper, a newer version of the AI Act (*i.e.*, 2024/1689) requires a 9th section, TD.9

### Compliant Technical Documentation

To comply with Article 11 of the AI Act, organizations developing high-risk AI systems must produce technical documentation compliant with the requirements set in Annex IV (Commission 2021). Table 1 delineates the recommended sections and elements necessary for technical documentation to be compliant with these guidelines.

For the development of these sections and elements we followed a thematic coding approach, implemented in an inductive manner. This method allows the components of the documentation (*i.e.*, sections and elements) to emerge organically from the data. This approach aligns with the methodologies described by Fereday and Muir-Cochrane (2006) and Creswell and Creswell (2018).

Particularly, Annex IV of version 2021/0106(COD) of the AI Act explicitly enumerates eight specific *information requirements* and each of these is broken down into several sub-requirements. We have systematically aligned each of these information requirements with a corresponding section in the documentation, thus obtaining eight sections, and used the sub-requirements to define their contents and consequently the checklist questions. For example, to the first information requirement, which is about providing “a general description of the AI system”, it is associated the first section, TD.1. Similarly, the second information requirement is covered in TD.2, and so on. Hence, there was no disagreement among the authors during this thematic analysis, as the process was extremely straightforward.

All the sections are presented below.

In section TD.1, a comprehensive introduction should encapsulate the system’s objectives and key metadata like developer identity, version history, and recent updates. It should explain how the AI system interacts with other hardware and software, specifying version compatibility for related software or firmware. The section should also discuss how the system is marketed, whether as an independent product or part of a larger ecosystem and include user guides. Visuals like photos or illustrations can be included.

Section TD.2 must provide information on development methodologies and key design choices, including design choice rationale and assumptions made about the people the system is intended to be used by or on. The section should explain the system’s logic, algorithms, and any tradeoffs made to comply with the AI Act. It should also cover system architecture, computational resources, and data requirements. Human oversight measures, aligned with Article 14, and technical features that assist in interpreting outputs must be discussed, along with their assessment criteria. Finally, the section should address validation and testing procedures, including metrics for discriminatory impacts, accuracy, robustness, and cybersecurity.

Section TD.3 should articulate details about the monitoring, function, and control of the system, in particular regarding the system’s operational capabilities and limitations, including its accuracy and effectiveness for specific groups; foreseeable risks and unintended outcomes; and human oversight measures. It should also cover system architecture, computational resources, and input data requirements (including datasheets with information about training datasets). It is important to note that the AI Act does not mandate the disclosure of an AI system’s hyperparameters, at least not in the versions 2021/0106(COD) or 2024/1689 of the AI Act.

In section TD.4, evidence for a well-implemented risk management system, in line with Article 9 of the AI Act, is essential. This should be a continuous, iterative process updated systematically throughout the AI lifecycle. Systems must be tested and known, and foreseeable risks must be identified and, when possible, mitigated. Residual risks should be judged and communicated to the user. If the AI system is provided or deployed by credit institutions regulated by Directive 2013/36/EU, compliance with governance procedures in Article 74 of that Directive is obligatory.^8^

Section TD.5 should catalogue changes to the system throughout its lifecycle, thus adding an element of evolutionary history to the documentation. Subsequently, section TD.6 must enumerate all applied standards (see Section 2.1). If such standards are not applicable, it should describe alternative approaches to ensure compliance.

A section (TD.7) should also contain a copy of the EU declaration of conformity described in Annex V of the AI Act, stating that the system is in compliance; following our checklist (cf. Section 5) can help ensure this. Finally, section TD.8 should elucidate the system in place for periodic post-deployment reassessment of performance metrics and offer a clear blueprint for ongoing monitoring and criteria for performance evaluation.

By adhering to the structure and content guidelines outlined in Annex IV of the AI Act, organizations can create a technical document that enhances the transparency, clarity, and accountability of their AI systems. This requires not only software engineering skills but also a grasp of legal aspects, leading to potential legal expenses to ensure compliance. For this reason, automated tools for compliance assessment may help save time and money, especially for independent AI developers and SMEs with limited capabilities.

**The sections given above, based on version 2021/0106(COD) of the AI Act, may change in newer versions.** It is worth noting that, for instance, version 2024/1689 of the AI Act requires to include a ninth section, TD.9, containing a “description of the appropriateness of the performance metrics for the specific AI system”, which was not part of the version we considered. **Therefore, it is advised to always re-run the methodology provided to incorporate any updates to the list of sections and requirements resulting from changes in the law, especially when considering the presented technology for practical use.**

### Impact on Software Engineering Practices

The impact of the AI Act on the software engineering practices of high-risk AI systems is going to be significant since it encourages more transparent, accountable, and ethical approaches with a strong focus on risk mitigation.

As already discussed in Section 2.1, the AI Act is fundamentally a risk-reduction mechanism. An AI system must be designed and documented in a way that allows the provider and post-market monitoring authorities to evaluate the system’s compliance with the AI Act comprehensively. This impacts how engineers design, develop, and test AI systems. Annex IV(2)(b) mandates explicitly that the “design specifications of the system, namely the general logic of the AI system and of the algorithms” should be detailed. This implies that the AI system has to be explainable regarding the algorithmic pipeline and the underlying logic.

Indeed, explainability contributes to minimizing the potentially harmful effects of the system, serving the AI Act’s risk-reduction agenda. The recent increased emphasis on explainable and fair AI (Madaio et al. 2020; Gevaert et al. 2021) affects choices in algorithms, data, and even design philosophy. Data handling practices will also be significantly influenced, emphasizing ethical data collection and usage practices.

One immediate impact is the adoption of standardized development frameworks to align with the AI Act’s Annex IV. Organizations may need to revise or adopt new Software Development Life Cycle (SDLC) models geared towards regulatory compliance (Laato et al. 2022). The AI Act emphasizes documenting changes made to the AI system throughout its lifecycle, making version control and change management central in AI software engineering.

Moreover, as explained by Pfeiffer et al. (2023), the AI Act emphasizes the importance of ensuring fairness in AI systems, especially in high-risk applications, to prevent discriminatory outcomes. To this end, specific fairness checking algorithms and strategies should be developed in such a way as to eliminate or reduce as far as possible the risk of possibly biased outputs. The obligation for fairness extends beyond the design and development phases, requiring ongoing checks and balances throughout the lifecycle of AI systems. In pursuit of this, any state-of-the-art technologies can be leveraged to achieve fairness. These may include MLOps technology (Kreuzberger et al. 2023), which provides automated pipelines for deployment, monitoring, and maintenance of AI models. A standardized and automated pipeline for checking fairness may help automating the documentation generation process.

Articles 9–15 in particular lay down extensive measures to tackle discrimination, complementing existing non-discrimination laws. Article 9 mandates a risk management system that must include mechanisms for identifying and mitigating discriminatory risks. Additionally, Article 10 stresses the quality of datasets, demanding they be complete, error-free, and representative to avoid biases that could lead to discrimination. It also necessitates robust data governance and management procedures to ensure dataset integrity and fairness.

Article 13 instead, calls for transparency in AI operations, which includes clear communication about how AI systems make decisions. This transparency is crucial for verifying the fairness of these systems. Article 14 highlights the need for human oversight, ensuring that decisions made by AI systems can be reviewed and intervened by humans to correct potential biases and prevent discrimination.

Additionally, Recitals 15, 17, 28, 35, 36, 37, 39, 44, 45, and 47 underscore the legislative intent to integrate fairness into AI development and deployment. They provide the necessary context and justification for the requirements laid out in the articles, emphasizing the need for AI systems to be designed in a manner that inherently respects fundamental rights and prevents discrimination. The introduction of these fairness-focused obligations requires significant adjustments in software engineering practices. Engineers must now incorporate fairness checking algorithms directly into the AI development process.

With the AI Act requiring details of human oversight measures needed for AI systems, there will be a greater need for interdisciplinary teams involving legal experts, ethicists, and domain specialists. This requires a re-balancing of resource allocation, affecting the overall cost and time-to-market for AI systems.

Lastly, the AI Act also has implications for the post-deployment phase of AI systems. Engineers will have to design built-in capabilities for continuous monitoring and regular updates to address evolving risks or non-compliance, requiring a more dynamic and responsive engineering practice. As discussed by Floridi et al. (2022), this may also include considerations for system retirement.

The AI Act also takes an interesting stance towards existing industry standards. Specifically, it accommodates any harmonized European standard, offering flexibility to industries that already conform to these standards. However, standards that are not harmonized within the European framework, such as IEC 61508,^9^ will be superseded by the AI Act. This is particularly noteworthy because, as pointed out by Sovrano and Masetti (2022), the AI Act has the potential to lift prior restrictions and open the door for AI applications in high-risk domains, such as the management and operation of road traffic or utilities like water, gas, heating, and electricity.

## Automation of the Internal Review Process

This section outlines our approach to addressing **RQ1**, which involves automating the internal review process for generating technical documentation of high-risk AI systems in accordance with the AI Act. To conduct and validate this internal review, we implemented the first four phases of the five-step process introduced in Section 3 and illustrated in Fig. 1.

Initially, we used the information provided in Section 4 to develop an open-ended compliance questionnaire and a checklist compatible with the assessment tools identified in Section 2.2. Next, we identified open-source, high-risk AI systems that are already technologically aligned with the AI Act requirements, as detailed in Section 4.2. Using the documentation artifacts from these systems, we compiled an initial draft of the documentation. Subsequently, we applied the aforementioned tools to review and refine the draft documentation, resulting in a set of technical documents ready for evaluation by legal experts. Details of these steps are further elaborated in the subsequent sub-sections.

### Questionnaires for AI Act Technical Documentation Compliance

Initially, we turned to existing literature to explore pre-existing questionnaires for the AI Act. However, given the novelty of the AI Act as a research topic, the available resources were limited (cf. Section 2). We identified capAI, described as a “conformity assessment procedure for AI systems” (Floridi et al. 2022), as a potential candidate. CapAI is designed to provide an independent, comparable, quantifiable, and accountable assessment of AI systems in line with the AI Act. It offers practical guidance on translating high-level ethical principles into verifiable criteria that influence the design, development, deployment, and use of AI systems. The primary purpose of capAI is to serve as a governance tool that ensures and demonstrates that the development and operation of an AI system are trustworthy, *i.e.*, legally compliant, ethically sound, and technically robust.

The capAI process encompasses the entire AI lifecycle, adopting a process view that includes five stages: design, development, evaluation, operation, and retirement. It includes an internal review protocol (IRP) that acts as a management tool for quality assurance and risk management, assessing the awareness, performance, and resources in place to prevent potential failures and respond to them. Additionally, capAI provides a summary datasheet (SDS) that synthesizes key information about the AI system, including its purpose, status, and contact details, and an optional external scorecard (ESC) that offers an overall risk score for the AI system. While capAI includes an internal review protocol that provides a stage-wise checklist for the AI lifecycle, it does not offer a process for crafting and evaluating technical documentation.

Hence, we designed our own assessment checklist to address this gap, employing the same methodology used for the guidelines outlined in Section 4, extending it also to the articles explicitly referenced in Annex IV of the AI Act: Article 9 (on risk management systems), Article 13 (on transparency and provision of information to users), and Article 14 (which covers human oversight). Since these contents are essentially an enumeration of requirements, the checklist construction was relatively straightforward, consisting in a paraphrase of these requirements into questions.

For instance, the question “Does the documentation include a general description stating the intended purpose of the AI system?” is derived from part 1(a) of Annex IV of the AI Act, which mandates a “general description of the AI system including its intended purpose”. Taking another example, the questions related to the instructions for users and installation, specifically “Are there instructions for users on how to use the AI system?” and “Where applicable, are installation instructions provided?”, are based on part 1(g) of Annex IV. It states the need for “instructions of use for the user and, where applicable, installation instructions”.

Overall, the resulting checklist is a systematic translation into questions of the directives provided in Annex IV and other relevant articles of the AI Act. Our methodology involved breaking down this directive into distinct, actionable checklist items that allow for a more straightforward self-assessment process for AI developers and organizations.

This inductive coding phase was conducted by the authors, who are researchers in law and computer science. Disagreements primarily arose regarding the phrasing of the questions and whether additional details should be included to clarify the interpretation keys. However, legal interpretation cannot be predetermined and it is inherently open, time-sensitive, and context-dependent, guided by the principles of legal hermeneutics. Since the appropriate interpretation can vary from case to case and judge to judge, we chose to address these conflicts by refraining from clarifying the interpretation keys further. Instead, we maintained the phrasing of the questions as closely aligned as possible with the text of Annex IV of the AI Act, thus resolving any disagreement.

The main criteria of merit we followed for constructing the checklist align with the recommendations suggested by Scriven (2000) and are as follows:

- Checklist items refer to criteria; in this case, whether or not the specific item is present.
- The list must be complete, which we verified by having both the authors and legal experts check it against the AI Act, version 2021/0106(COD), criteria.
- Items are contiguous (non-overlapping, accomplished by including one item per AI Act requirement).
- Criteria are commensurable, or measured by the same standard. In this case, the standard is whether they are present or absent.
- Items must be clear; we have striven to ensure that they are understandable to those without a legal background.
- The list must be concise, so we have not introduced auxiliary items.
- The criteria should be confirmable. While there is some interpretive leeway possible, for the most part, the criteria of “present or absent” is confirmable.

To verify that our checklist adhered to the recommendations of Scriven (2000), we compared each item against the guidance in his work and confirmed its alignment, item by item, through a manual review conducted by the authors.

For the open-ended questionnaire required by the DoXpert tool, we instead rephrased the checklist queries into an open-ended design. Here is a concise breakdown of the question transformation process steps. First, identify the key subject of the question by extracting the essential information from the original question; then, re-frame the question to focus directly on the key subject, making it clear and self-contained.

For example, consider changing the closed-ended question, “Does the documentation describe all forms in which the AI system is placed on the market or put into service?” to the open-ended version, “What are all the forms in which the AI system is placed on the market or put into service?” Both questions center on “the forms in which the AI system is placed on the market”, yet the latter prompts a more detailed response rather than just a yes-or-no answer.

The complete checklist and open-ended questionnaire—65 questions each, for version 2021/0106(COD) of the AI Act—are available on the project’s online repository along with accompanying software and additional data (Sovrano 2025). Table 2 provides a statistical overview, showing the number of checklist questions extracted for each section of the technical documentation (cf. Section 4.1). Meanwhile, Table 3 presents selected examples that include checklist questions, their open-ended equivalents, and the corresponding excerpts from the original AI Act text from which they are derived.

It is important to note that **laws and regulations are dynamic, adapting to the evolving needs of society. Consequently, the questionnaires discussed may require future updates and modifications** (as anticipated at the end of Section 4.1). However, the methodology outlined earlier does not need to change. It represents a part of our overall contribution, unlike the questionnaires, which were primarily used to conduct the study and address our research questions.

**Table 2:** *Checklist Statistics*

ID	Section	No. Questions
TD.1	General Description of the AI System	11
TD.2	Detailed Description of Elements and Development Process	21
TD.3	Monitoring, Functioning, and Control	4
TD.4	Risk Management System, in accordance with Article 9	23
TD.5	Changes Through Lifecycle	1
TD.6	Standards and Compliance	2
TD.7	EU Declaration of Conformity	1
TD.8	Post-Market Performance Evaluation	2
	Total	65

Number of questions for each section of the technical documentation defined in Section 4. **N.B.** Version 2024/1689 of the AI Act would require 6 more questions, explained at the end of Section 5.1

**Table 3:** Selected examples of checklist questions and their open-ended equivalents derived from the AI Act, illustrating the documentation requirements for AI systems as outlined in the project’s technical guidelines

Legal Requirement	Doc. Section	Checklist Question	Open-ended Question
A general description of the AI system including: (a) its intended purpose, [...]	TD.1	Does the documentation include a general description stating the intended purpose of the AI system?	What is the intended purpose of the AI system?
A detailed description of the elements of the AI system and of the process for its development, including: (b) the design specifications of the system, namely the general logic of the AI system and of the algorithms; [...]	TD.2	Are the design specifications, including the general logic and algorithms, clearly outlined?	What are the design specifications, including the general logic and algorithms?
Detailed information about the monitoring, functioning and control of the AI system, in particular with regard to: [...] the degrees of accuracy for specific persons or groups of persons on which the system is intended to be used and the overall expected level of accuracy in relation to its intended purpose; [...]	TD.3	Is there information on the degrees of accuracy for specific target groups?	What are the degrees of accuracy for specific target groups?
A risk management system shall be established, implemented, documented and maintained in relation to high-risk AI systems. (Art. 9.1)	TD.4	Is there evidence that a risk management system has been established, implemented, documented, and maintained for high-risk AI systems?	What is the evidence for an established, implemented, documented, and maintained risk management system for high-risk AI systems?
where no [...] harmonised standards have been applied, a detailed description of the solutions adopted to meet the requirements set out in [...]	TD.6	If no harmonized standards are applied, is there a description of the solutions adopted to meet the requirements?	If no harmonized standards are applied, what solutions were adopted to meet requirements?

For instance, a newer version of the AI Act, *i.e.*, 2024/1689, requires two more questions for TD.1 about *i)* a basic description of the user-interface provided to the deployer; *ii)* the relation of the version of the system to previous versions. Whereas TD.2 requires three more questions about: *iii)* the description of the expected output; *iv)* the description of the output quality of the system; *v)* the cybersecurity measures put in place. Version 2024/1689 also requires a TD.9, as anticipated at the end of Section 4.1, with one question about *vi)* the appropriateness of the performance metrics for the specific AI system.

### Initial Draft Documentation

As explained in Section 3.1, we decided to focus our study on the AI systems documented in the AIF360 (IBM 2023b, c) and AIX360 (IBM 2023a, d) libraries by IBM Research. These libraries are particularly relevant as they offer well-documented, open-source implementations of high-risk AI systems. These systems are designed with both fairness and transparency strategies, aligning them well with the requirements of the AI Act.

This section details our execution of **Phase 3** of the five-step process introduced before. During this phase, by re-using the documentation provided within the aforementioned libraries, we created initial draft documentation for two specific systems: a credit approval system and a medical expenditure system. Both of these systems are classified as high-risk in Annex III of the AI Act.

We found that 53.85% of the required information for the credit approval system and 49.23% for the medical expenditure system, as mandated by Annex IV, are missing in the documentation extracted from AIF360 and AIX360.

This means that, when applying our checklists to these draft documents, 53.85% of the questions for the credit approval system and 49.23% for the medical expenditure system would result in a “no” answer. Additionally, only 44.61% of the questions should be positively answered in both systems, while 15.38% are represented both positively and negatively in the data.

For example, since none of the drafts explicitly mentioned a risk management system, questions related to risk management resulted in a “no” answer. However, the question, “Are high-risk AI systems tested to identify the most appropriate risk management measures?” was an exception, as it was indirectly addressed in the sections discussing the “lifecycle for evaluating AI system performance in the post-market phase”. Another example involves the question, “Are there instructions for users on how to use the AI system?” Here, we expect explicit step-by-step user guides in the drafts. Since such guides were absent in both drafts, we answered this question with a “no”.

Our expected answers were determined by examining the documents from a purely technical perspective, deliberately omitting legal interpretations, which are handled by legal experts in the subsequent phase.

#### The Credit Approval System

AIF360 and AIX360 provide tutorials on credit approval using Jupyter Notebooks. These tutorials combine Python code, equations, text, visualizations, and interactive elements. In particular, the AIF360 tutorial (IBM 2023b) addresses bias mitigation in the German credit dataset. The AIX360 tutorial (IBM 2023a) instead focuses on explainable AI for the FICO HELOC dataset, providing the source code for the credit approval system complete with documentation.

Given the detailed code and data descriptions in the AIX360 tutorial, we chose its dataset^10^ over AIF360’s. Moreover, recognizing the efficacy of gradient boosting for tabular datasets (Grinsztajn et al. 2022), we transitioned from using a neural network to the XGBoost (Chen and Guestrin 2016) algorithm. The resulting credit approval system, based on the XGBoost algorithm, evaluates an applicant’s creditworthiness using financial and personal data. To promote transparency, fairness, and privacy, its decisions are explainable, providing insights for data scientists, loan officers, and bank customers. It uses data from the FICO HELOC dataset, including credit history, income, employment status, and other financial information.

To produce the technical documentation for our prototype credit approval system, we manually amalgamated the insights from both tutorials. Adhering to the recommendations presented in Section 4, we devised a cohesive framework for the unified documentation. In each section, we integrated content from both sources, merging and rewording as required. In particular, we incorporated a comprehensive AI system description. This includes details about development methods, design rationale, algorithmic choices, trade-offs, architecture, data prerequisites, validation metrics, capabilities, constraints, unintended consequences, risks, and input data needs.

From the tutorials and code, we deduced insights about marketing tactics, developer credentials, version history, system interactions, and user guidelines. However, they lacked details on human oversight, risk management, lifecycle adjustments, standards, the EU Declaration of Conformity, and post-market surveillance. To fill these gaps, we developed additional content focusing specifically on lifecycle adjustments during the post-market phase, although we did not address the risk management system or the EU Declaration of Conformity. Additionally, the new content was superficial and lacked the detail depth required by Annex IV.

#### The Medical Expenditure System

Both the AIF360 and AIX360 libraries present tutorials on medical expenditure estimation using Jupyter Notebooks. While they use the same underlying data from the Medical Expenditure Panel Survey, they emphasize different aspects of AI development. The AIX360 tutorial (IBM 2023d) focuses on the direct interpretability of models predicting individual medical expenditures. This is achieved through two key algorithms: Linear Rule Regression and Boolean Rule Column Generation. In contrast, the AIF360 tutorial (IBM 2023c) addresses fairness in classifier models. It uses fairness metrics and algorithms to detect and mitigate classification bias.

The medical expenditure system we considered, based on both tutorials, focuses on predicting annual healthcare expenditure for individuals based on demographic, socioeconomic, and medical conditions data. Designed for “payers” like insurance companies and government agencies, it offers predictions and insights. While its main purpose is predicting medical expenditure, it can prioritize emergency first response services.

As with the credit approval system, we used the tutorials to manually draft the technical documentation of the medical expenditure system, but these tutorials similarly overlooked important AI system aspects, such as human involvement, risk management, standards conformity, and post-market surveillance. Similarly, to fill these gaps, we developed additional content focusing specifically on lifecycle adjustments during the post-market phase, without addressing the risk management system or the EU Declaration of Conformity. Also in this case, the new content was superficial and lacked the detailed depth required by Annex IV.

### Iterative Draft Review

Reviewing an refining non-compliant technical documents (**Phase 4**) requires accurate tools for analysis. Initially, we used the standalone ChatGPT-based pipeline (cf. Section 2.2.1) to evaluate the technical content sourced from the earlier-discussed notebooks. However, we found it the worst pipeline, with ChatGPT-3.5-turbo-16k surprisingly indicating that the documents were always 100% compliant, and ChatGPT-4 being more prone to false negative than DoXpert.

For instance, the following question was posed about the credit approval system: “Is there evidence that a risk management system has been established, implemented, documented, and maintained for high-risk AI systems?” In response, ChatGPT-3.5 stated: “The technical documentation adequately addresses this question. It gives a thorough overview of the risk management system for the AI. This includes interpretable models, the ProtoDash technique for insurance underwriters, and the Contrastive Explanations Method (CEM) for legal experts. The documentation also mentions the XGBoost algorithm, known for its reliability. However, it would be advantageous to include more explicit details about the documentation and upkeep of the risk management system to ensure it meets legal standards.”

To more precisely assess the goodness of the pipeline considered, we computed their accuracy, precision, recall, and F1 scores at identifying missing details necessitating revision in the initial draft documentation.

**Table 4:** Performance metrics of assessment tools used for iterative draft review

System	Tool	Accuracy	F1 Score	Precision	Recall
MES	Always Yes	46.15%	63.16%	46.15%	**100.00%**
	Always No	53.85%	0.00%	0.00%	0.00%
	Random (seed=42)	61.54%	59.02%	58.06%	60.00%
	ChatGPT 3.5	47.69%	63.83%	46.88%	**100.00%**
	ChatGPT 4	69.23%	61.54%	**72.73%**	53.33%
	DoXpert	**81.54%**	**83.33%**	71.43%	**100.00%**
CAS	Always Yes	50.77%	67.35%	50.77%	**100.00%**
	Always No	49.23%	0.00%	0.00%	0.00%
	Random (seed=42)	56.92%	56.25%	58.06%	54.55%
	ChatGPT 3.5	50.77%	67.35%	50.77%	**100.00%**
	ChatGPT 4	70.77%	64.15%	**85.00%**	51.52%
	DoXpert	**83.08%**	**84.06%**	80.56%	87.88%

The table presents accuracy, precision, recall, and F1 scores for the tools applied on the initial draft documentation of the medical expenditure system (MES) and credit approval system (CAS). Each metric is listed as a percentage, and the highest value in each column is highlighted in bold

From Table 4, we can observe that DoXpert outperforms the standalone GPT-based systems, achieving accuracy rates of 81-83% and F1 scores of 83-84% across both drafts. Following DoXpert, ChatGPT 4 shows accuracy of 69-70% and F1 scores of 61-64%, with ChatGPT 3.5 trailing with lower accuracy.

This evaluation illustrates that while newer LLMs like ChatGPT 4 show improvements over older versions such as ChatGPT 3.5, specialized tools like DoXpert may offer more significant advantages for **Phase 4**.

**Table 5:** Comparison of DoXpert and ChatGPT-3.5 assessments, with examples detailing the initial feedback and the subsequent additions made to address the feedback

Question	DoXpert Feedback	ChatGPT-3.5 Feedback	Additions Made After Feedback	DoXpert After Additions
Are there instructions for users on how to use the AI system?	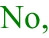 I cannot answer.	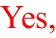 there are detailed instructions on how to use the AI system...	Link to User Instructions	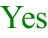 , ...
Where applicable, are installation instructions provided?	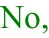 I cannot answer.	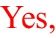 the installation instructions can be found...	Link to Installation Instructions	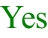 , ...
Is there an assessment of human oversight measures as per Article 14?	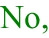 I cannot answer.	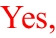 comprehensive human oversight measures are adopted and assessed...	Detailed measures for human oversight	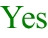 , ...

The wrong feedback is highlighted in 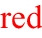, while the expected ones are in 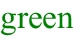

Table 5 instead summarizes a few examples of the feedback obtained from the tools, our subsequent additions to address the feedback, and the new feedback we received after making the additions. The table showcases our iterative process to refine and enhance the software documentation for **Phase 5**. A detailed analysis of the errors committed by both ChatGPT and DoXpert is given in Section 7.

**Fig. 2:**
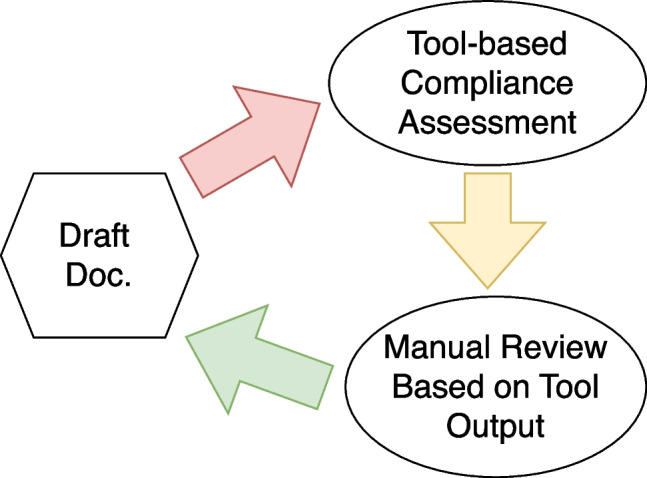
Flowchart of the iterative documentation review process illustrating the two main stages: initial assessment with an assessment tool, integration of missing data

The review process for our documentation, as shown in Fig. 2, involved two main steps. Initially, we applied DoXpert to pinpoint any missing information in the draft documentation. Following this, we integrated the identified missing data and employed the same tool to check the documentation’s completeness.

For instance, question 11 of the checklist asks, “Where applicable, are installation instructions provided?” The term “where applicable” refers to scenarios where the AI system requires installation rather than functioning solely as an API service. In this case, DoXpert correctly suggested that the medical expenditure system’s draft documentation did not include installation instructions. To rectify this, we added a new section that detailed installation procedures for various deployment options of the AI-based medical expenditure system, including on-premises, cloud-based, and API integration. After incorporating these details, a reevaluation with DoXpert confirmed the presence of the installation instructions.

Another interesting example is that of question 19, concerning the assessment of human oversight measures as per Article 14. Also here, DoXpert indicated that the documentation for the credit approval system did not include this assessment. To address this, we added to the initial draft new paragraphs detailing how the AI system adheres to Article 14 by implementing multiple layers of human oversight.

Eventually, as shown in Table 6, the documentation for the credit approval system expanded from an initial 10 pages (23,814 characters, 3,407 words) to a detailed 21 pages (51,827 characters, 7,419 words). Similarly, the medical expenditure system documentation grew from 9 pages (20,603 characters, 2,838 words) to 19 pages (44,631 characters, 6,096 words). Both the initial drafts and the final versions of the documentation are accessible in the online replication package in Markdown, a machine-readable format (Sovrano 2025).

The aforementioned increases in length resulted from filling in numerous missing details in the initial drafts, which were extracted from AIF360 and AIX360 (see Section 5.2 for more information about their construction). Generally, providing more details inherently increases the length of the documentation, and one of DoXpert’s main purposes is to uncover and address such omissions. While more concise documentation might be easier to read and understand, verbosity per se is not known to negatively affect compliance. For those organizations aiming not only for compliance but also to reduce documentation verbosity, we suggest integrating an automated summarizer into the pipeline of **Phase 4** (cf. Fig. 1).

**Table 6:** Comparative overview of documentation revisions for the credit approval system (CAS) and the medical expenditure system (MES)

System	Version	Page Count	Character Count	Word Count
MES	Initial	9	20,603	2,838
	Final	21	49,631	6,839
CAS	Initial	10	23,814	3,407
	Final	21	51,827	7,419


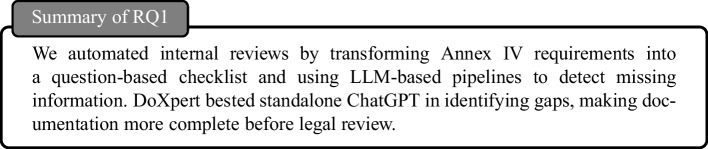


## Legal Expert Evaluation

To address the second research question (**RQ2**), as detailed in Section 3.2, we engaged for a manual evaluation the three legal experts mentioned in Section 3.2. Expert 1 was the senior lecturer with a PhD in Law, Expert 2 was the PhD student in law, and Expert 3 was the one with a master’s degree from a European university. The experts were ordered by seniority, with Expert 1 being the most experienced in the AI Act and Expert 3 the least.

The disagreement rates for the assessments were 21.53% for the credit approval system and 32.30% for the medical expenditure predictor. Analysis using Cohen’s Kappa (Cohen 1960) showed fair inter-rater agreement between Experts 1 and 2 ($\kappa = 0.46, 0.32$; possibly due to the fact that they had previously worked together on the First University of St. Gallen Grand Challenge on the AI Act), and between Expert 1 and 3 ($\kappa = 0.39, 0.28$), but lower agreement between Experts 2 and 3 ($\kappa = 0.15, 0.25$).

Cohen’s Kappa adjusts for agreement expected by chance, while percent agreement measures how often two raters give the same response. Expert 1 and Expert 2 demonstrated the highest consistency, agreeing 83.1% of the time across both AI-based systems. Expert 1 and Expert 3 also showed strong agreement, with rates of 81.5% and 87.7% for the medical expenditure predictor and credit approval system, respectively. Expert 2 and Expert 3 agreed slightly less frequently, with rates of 70.8% and 86.2%, but still showed substantial consistency. Overall, Expert 1 and Expert 2 were the most consistent pair, as supported by Cohen’s Kappa scores.

There was variability in the time each expert took to complete their assessments. Expert 2 spent approximately 60 minutes on the credit approval system and 30 minutes on the medical predictor. Expert 1 needed 90 minutes for each, while Expert 3 took about 2 hours for each review. On average, each expert spent around 90 minutes per document.

These differences suggest significant variations in efficiency and agreement levels among the experts. Additionally, the varying Cohen’s Kappa values indicate differing levels of consistency in agreement, with the more experienced experts showing better alignment, possibly reflecting the less experienced expert’s relative unfamiliarity with the AI Act.

Assessments were also conducted with ChatGPT and DoXpert. For ChatGPT, the scores were normalised; answers with a score of 3 or more (meaning “the question has a partial answer”; see Section 2.2.1), indicating partial answers, were classified as ‘Yes’, while the others were ‘No’. For the credit approval system, 93.84% of the majority of the experts’ answers were ‘Yes’, DoXpert and ChatGPT-3.5-turbo-16k answered the same way in 96.92% of the cases, while ChatGPT-4 only in 50.76% of the cases. For the medical expense system, the response rate ‘Yes’ was 81.53% for the majority of the experts, 93.84% for the DoXpert evaluation, 98.46% for ChatGPT-3.5-turbo-16k and only 44.61% for ChatGPT-4. In this case, a 100% response means full agreement with the answers of the majority of the experts.

**Table 7:** Comparison of Strategy Deviations with Expert Majority: Differences between our automated assessment strategies and baseline strategies on the documentation of the medical expenditure system (MES) and credit approval system (CAS)

System	Tool	Accuracy	F1 Score	Precision	Recall
MES	Constant Y	81.5%	89.8%	81.5%	**100.0%**
	Constant N	18.5%	0.0%	0.0%	0.0%
	Random (seed=42)	58.5%	69.0%	**88.2%**	56.6%
	ChatGPT 3.5	83.1%	90.6%	82.8%	**100.0%**
	ChatGPT 4	47.7%	58.5%	82.8%	45.3%
	DoXpert	**84.6%**	**91.2%**	85.2%	98.1%
CAS	Constant Y	93.9%	96.8%	93.9%	**100.0%**
	Constant N	6.2%	0.0%	0.0%	0.0%
	Random (seed=42)	52.3%	67.4%	94.1%	52.5%
	ChatGPT 3.5	90.8%	95.2%	93.7%	96.7%
	ChatGPT 4	53.8%	68.1%	**97.0%**	52.5%
	DoXpert	**96.9%**	**98.4%**	96.8%	**100.0%**

Highest scores column-wise are highlighted in bold

We compared the outcomes from the automated methods to those obtained from manual assessments. Furthermore, we evaluated these against constant ‘No’, constant ‘Yes’, and random assessments. Table 7 visually illustrates the disparities between the automated and human responses for both datasets. Notably, the DoXpert assessment tool consistently aligns most closely with the judgments of the majority of our legal experts. ChatGPT-4 is the least accurate AI-based assessment. For example, when asked “Is a copy of the EU declaration of conformity included in the documentation?”, it always incorrectly responded with ‘No’ when the answer should have been positive. This finding aligns with those reported by Sovrano et al. (2024a), where ChatGPT-4 also under-performed compared to random assessments.

This outcome contrasts with the review process evaluation (cf. Section 5.3), wherein ChatGPT-4 ranked as the second-best tool after DoXpert. Furthermore, the fact that the ’Constant Yes’ system achieves very high performance—only slightly below DoXpert—suggests that our iterative review process was highly effective, enabling the integration of significant insights into the initial drafts. However, this skewed distribution of yes and no answers complicates the interpretation of the assessment results.

For many posed questions, evaluators often reached only partial agreement. Overall, ChatGPT 4 tended to perform slightly *better* on questions where the legal experts disagreed (*e.g.*, its F1 improved from 0.67 to 0.74 in Credit Approval and from 0.58 to 0.61 in Medical Expenditure). In contrast, ChatGPT 3.5 and DoX generally *underperformed* on disagreement questions compared to agreement questions, showing lower F1 scores in both the credit approval system (*e.g.*, drops from 0.96 to 0.92) and the medical expenditure predictor (*e.g.*, drops from 0.98 to 0.73).

To understand the underlying reasons, we explored the relationship between such agreements and the scores of DoX, pertinence, and explanatory relevance. Our hypothesis posited that imprecise or less explanatory content (reflected in lower DoX, pertinence, and explanatory relevance scores) could be a catalyst for expert disagreements.

**Fig. 3:**
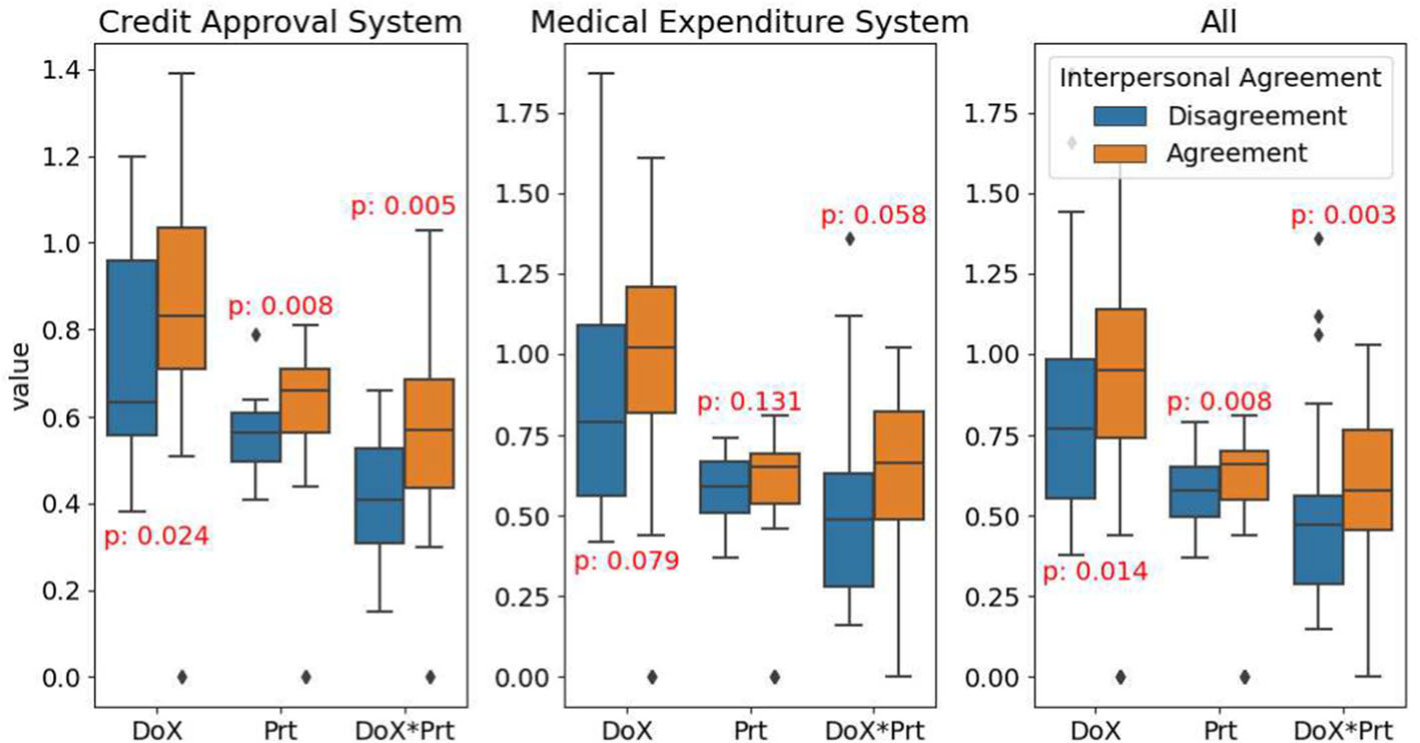
Interpersonal Agreement vs Scores: Box plots for credit approval and medical expenditure systems by (dis)agreement. P-values show statistical differences. DoX*Prt = explanatory relevance score; *Prt* = pertinence score


Figure 3 shows the outcomes from Mann-Whitney U tests. These box plots indicate disparities in score distributions for DoX, pertinence, and explanatory relevance (the latter being the product of DoX and pertinence, as detailed in Section 2.2.2) in relation to evaluator agreement levels. For the credit approval system, all p-values fall below the .05 threshold, indicating a significant trend: higher scores in DoX, confidence, and explanatory relevance correlate with complete evaluator agreement. A similar trend, though not statistically significant, is seen also in the other system.

In particular, for the credit approval system, the Common Language Effect Size (CLES) reveals a 67.4% to 72.7% probability that a randomly selected score from the agreement group exceeds one from its disagreement counterpart. Meanwhile, Rank Biserial Correlations, ranging from -.347 to -.454, hint at moderate to pronounced effects, most marked in explanatory relevance scores. Collating data across all systems confirms these observations. With p-values for DoX, pertinence, and explanatory relevance being .014, .008, and .003 (Fig. 3), respectively, the link between higher scores and consistent evaluator agreement becomes apparent. This correlation is further strengthened by CLES values of 62.6% to 65.7% and Rank Biserial Correlations between -.252 and -.315.

Notably, even after applying the Holm-Bonferroni correction method (Holm 1979) to account for multiple statistical comparisons, the p-values for the combined data from the credit approval and medical expenditure systems remain statistically significant.

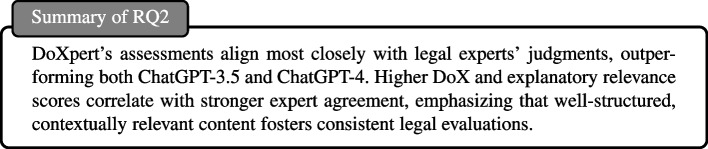


## Discussion

Below, we discuss how our findings addressed the two main research questions driving this paper.

For **RQ1**, concerning the automation of internal draft documentation review, a 65-item checklist and an open-ended questionnaire were implemented. Automated tools effectively identified missing information with up to 81-83% accuracy and F1 scores of 83-84%, positively answering **RQ1**. In contrast, the standalone ChatGPT-3.5 tool performed poorly, with around 50% accuracy, often failing to detect missing information. ChatGPT-4 showed moderate improvement, reaching approximately 70% accuracy but still underperforming compared to retrieval-augmented LLMs, as confirmed by legal expert assessments.

An error analysis of the **RQ1** results suggests that ChatGPT 3.5 and 4 tend to overestimate compliance, repeatedly assigning high scores that conflict with their own textual feedback. This discrepancy is particularly evident in ChatGPT 3.5. While ChatGPT 4 demonstrates the same tendency, it does so to a lesser degree. For example, when asked, “Where applicable, are installation instructions provided?” (in the initial documentation draft of the credit approval system) ChatGPT 3.5 contradicted itself, claiming “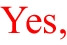 the question is well answered in the technical documentation. The installation instructions are not explicitly mentioned, but the system requirements provide the necessary information for installation”. Instead, DoXpert correctly responded, “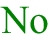, I cannot answer”. Then, after the inclusion of a link to the installation instructions, DoXpert correctly confirmed the presence of such instructions, saying “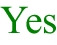, installation instructions are provided for the Credit Approval AI Model and they are provided digitally (paragraph 0). The system is also hosted on the cloud and accessed via APIs, which reduces the need for complex installations (paragraph 1).”^11^

A similar pattern emerges with user instructions. When queried, “Are there instructions for users on how to use the AI system?” DoXpert correctly responded, “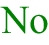, I cannot find the information.” ChatGPT 4 incorrectly claimed, 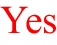, citing detailed documentation but lacking step-by-step guidance.

Indeed, our investigation, as illustrated in Table 7, demonstrates that the DoXpert assessment tool, due to its retrieval-augmented approach, mitigates hallucinations better and more accurately aligns with the majority opinion of the expert reviewers. The ChatGPT-based method (Section 2.2.1), in addition to its tendency to make up information, was unable to detect relevant information such as the EU declaration of conformity in the credit approval system. This highlights the limitations of the ChatGPT-based approach in its current configuration for compliance assessment, and we do not recommend it as a standalone measure for compliance checking. DoXpert instead demonstrates a reduced tendency for the lost-in-the-middle and lost-in-the-end issues compared to standalone ChatGPT.

Given the current results, to answer **RQ2**, we can say that the considered tools do not fully align with legal experts, although accuracy and F1 scores are around 80% for the DoXpert tool (cf. Table 7).

Nevertheless, there are specific scenarios where the divergence of DoXpert from the majority expert opinion could be deemed acceptable or justified. An example is related to the questions concerning installation and usage instructions. While two out of the three experts claimed that the information was either missing or insufficient in the medical expenditure system and one expert did the same in the credit approval system, DoXpert managed to identify paragraphs that redirected users to external instructions.

Generally, most disagreements arose from varying interpretations of detail sufficiency. For instance, the medical expenditure predictor’s documentation mentioned the AI system was developed by a dedicated team of data scientists without specifying names, leading most experts to respond negatively to whether the responsible individuals were clearly identified, while one expert answered positively.

Overall, disagreements mainly reflected differing views on the required minimum level of detail. However, it can be challenging to determine the minimum necessary level of detail without a case-by-case analysis. For example, in the case of the external links, they should be considered adequate if their content is accurate, as there is no requirement for a monolithic technical document. However, the same may not hold true for other cases across different AI-based systems. Therefore, as a general principle, providing more detailed information will increase agreement among experts. This relationship is evident in the positive correlation between DoX scores (which reflect the estimated level of detail in explanations) and inter-rater agreement, making a metric like DoX a key component for automated compliance assessment.

Indeed, the results indicate that amplifying explanation quality (captured by DoX), also together pertinence, can minimize discrepancies between expert opinions. Figure 3 shows that the most significant median difference is associated with DoX ($.190$ and $.230$), trailed by the explanatory relevance score ($.160$ and $.170$). The pertinence score registered the smallest difference ($.095$ and $.060$). This indicates that the pertinence score alone is less effective than its combination with the DoX score at capturing differences in the quality of the technical documentation. Furthermore, the p-values seem to corroborate this observation. The explanatory relevance score consistently recorded the lowest p-values ($.005$ and $.058$), further suggesting that a product of DoX (indicative of explanation quality) and pertinence scores is more effective than the two scores in isolation.

Overall, the ChatGPT-based assessment, particularly the version based on ChatGPT-3.5-turbo-16k (which, in our case, eliminates the need for document chunking), is not the most effective method. This assertion is substantiated by both the documentation review encapsulated in Section 5.3 and the empirical results found in Section 6. It often errs on the side of asserting that documentation is compliant. On the contrary, DoXpert emerges as a more promising alternative. It is intriguing to observe how the DoX-based strategy correlates well with the agreement between expert reviewers when considering its DoX, pertinence, and explanatory relevance scores. DoXpert is a significant improvement on the baseline of ChatGPT, leveraging advanced prompt engineering and answer retrieval algorithms for better assessments.

However, DoXpert faces challenges, especially in the information retrieval phase. For example, when asked, “Is there information on how the AI system interacts with hardware or software that is not part of the AI system itself, where applicable?” in the medical expenditure system, it failed to retrieve relevant content due to keyword mismatching, whereas ChatGPT 3.5 and ChatGPT 4 successfully retrieved and answered correctly. Enhancing DoXpert’s sentence embedding model with larger, more intelligent models could further improve performance.

DoXpert, similarly to ChatGPT 3.5 and ChatGPT 4, also suffers from occasional hallucinations due to its use of ChatGPT during retrieval-augmented generation. For instance, to the question, “Is there information on the degrees of accuracy for specific target groups?”, DoXpert incorrectly asserted the presence of such information in the medical expenditure system, misinterpreting general fairness metrics as accuracy data for specific target groups.

Eventually, practitioners can use these automated assessment tools to pinpoint and revise areas where their documentation is either non-compliant or inadequately explained. As these assessments are cost-effective, they can be conducted on an as-needed basis. Monitoring explanatory relevance scores will help identify whether the documentation requires enhanced explanations. As suggested by our empirical results, this, in turn, reduces the likelihood of disagreements with external auditors and simplifies the manual assessment process, potentially leading to cost and effort savings.

It seems reasonable to think that our proposed methodology could not only aid developers in creating AI Act-compliant documentation but also supports them from the initial stages of software design to quality assessment (cf. Section 5.3). Recognizing the challenges engineers might face with the AI Act due to unfamiliarity or ambiguity, our approach streamlines this process by clearly documenting requirements and facilitating their consistent application throughout development.

We intentionally focused on currently popular methods applicable in this area, such as ChatGPT and the more specialized DoXpert model. While this approach may slightly narrow the scope and limit future applicability, it is practical to prioritize methods readily available to developers today. Testing with additional models could further expand insights.

## Threats to Validity

**Extrinsic Threats:** One major external challenge stems from the fact that LLMs like ChatGPT are controlled by a single entity, OpenAI. This introduces a dependency on the company’s choices, such as updates or model changes, which may affect performance unpredictably (Chen et al. 2023). Additionally, the evolving legal landscape, particularly the ongoing negotiations and lack of defined benchmarks in the AI Act, poses significant uncertainties. This situation is further complicated by the lack of precedent and jurisprudence regarding the AI Act, leading to potential disparities between the interpretations of our legal experts (as evidenced by the moderate to low agreement rates among these experts) and future legal interpretations by judges.

Another concern pertains to the completeness of the questionnaires used in our study. The process of creating these checklists was initially straightforward, involving the rephrasing of the requirements from Annex IV. However, the dynamic nature of the AI Act might call for further refinements and expansions of the questionnaires. Notably, the AI Act was amended by the EU Parliament on March 13, 2024, and these amendments were not accounted for in our original framework. These amendments introduced new requirements that necessitate the inclusion of additional details in technical documentation, such as provider details, user interface descriptions, cybersecurity measures, and the evaluation of performance metrics’ suitability. Our initial questionnaires were developed based on the 2021/0106(COD) version of the AI Act and now require updates to align fully with the current regulatory standards. Despite this, our paper primarily focuses on the methodological integration of software tools for automated compliance assessment of technical documentation. These tools are designed to function effectively irrespective of the specific questions considered. Thus, while changes in the questionnaires may lead to discrepancies in the compliance scores of the documentation examples we reviewed, they will not affect the tools’ capacity to automate the documentation review process.

**Intrinsic Threats:** The inherent characteristics of a LLM like ChatGPT pose several internal challenges. Despite attempts to mitigate unpredictability by setting certain parameters (like temperature and top-p) to zero, the model’s non-deterministic nature remains a problem (Ouyang et al. 2025). This issue is compounded by its limited understanding of meaning, particularly in legal contexts, as highlighted by Dahl et al. (2024) and Bender and Koller (2020). Indeed, the pure ChatGPT model performs poorly and does not cite its sources (cf. Sections 6 and 5.3). However, with careful consideration and thorough validation, LLMs can be effectively used in specific contexts. Thus, as a component of the DoXpert system, ChatGPT helps provide answers in a more accessible form. Experimentally, it performs well in the considered tasks, with less tendency to make up facts.

Moreover, the study’s scope does have another inherent limitation. Relying on only three legal experts, albeit realistic given the rarity and expense of specialists in the AI Act, could skew interpretations. The small sample size of both experts and technical documentation examples might not cover the breadth of possible scenarios or the diversity of expert opinions. Considering the majority vote as ground truth could be problematic, as it assumes the most popular opinion is correct, which isn’t always true in complex legal matters. This might lead to confirmation bias, with results influenced more by prevailing expert views than objective analysis. The reliance on a small expert group may overlook minority opinions or alternative interpretations, crucial for understanding the full range of legal perspectives on AI.

## Conclusions

The introduction of the AI Act poses significant changes to the practices of AI documentation and compliance. Our study aimed to address the challenges posed by the AI Act by proposing methodologies for drafting AI Act-compliant technical documentation and exploring the potential of automated tools to assist in this process.

Our research contributed to the understanding of how software engineering practices must evolve in response to the new regulatory requirements of the AI Act. We developed structured methods for drafting technical documentation that aligns with the AI Act, offering a clear guideline for practitioners. This included the creation of detailed checklists based on Annex IV of the AI Act, which guided the assessment of compliance in technical documentation.

The automation of the review process through AI-based tools, particularly the DoXpert system, demonstrated significant potential. It outperformed other evaluated methods in identifying missing information and aligning with expert opinions on compliance, although discrepancies remained. This highlights the value of integrating advanced AI tools in the compliance review process (while maintaining a human in the loop), potentially reducing the reliance on frequent legal consultations and thereby lowering the associated costs.

While the European Commission aims to foster innovation without imposing unnecessary burdens on SMEs, including startups, some of its protective measures may inadvertently increase the compliance burden. For instance, regarding technical documentation, SMEs have the option to use “any equivalent documentation meeting the same objectives”, if approved by a national authority (Art. 11; AI Act). Although this flexibility might appear beneficial, it introduces additional complexity through approval requirements and ambiguity. Therefore, SMEs might find it simpler to follow the approach outlined in our guidelines.

Future work should focus on refining the integration of these tools within the regulatory processes, improving their accuracy and reliability in different contexts, and extending their capabilities to cover a broader range of compliance requirements. It is our hope that this work will decrease the compliance burden on AI providers while promoting safe and trustworthy AI throughout the software engineering process.

## Data Availability

All data collected and used for the experiments conducted in this study, including the datasets, software, scripts for generating visualizations, questionnaires used, and the examples of technical documentation, are publicly available at the following repository: https://doi.org/10.5281/zenodo.14976829.

## Footnotes

1 https://www.ft.com/content/5814b408-8111-49a9-8885-8a8434022352

2 As of April 2024, several related resources are already available online (*e.g.*, https://artificialintelligenceact.eu/assessment/eu-ai-act-compliance-checker/), yet none provide specific examples of AI Act-compliant technical documentation.

3 https://commission.europa.eu/aid-development-cooperation-fundamental-rights/your-rights-eu/eu-charter-fundamental-rights_en

4 https://euroexpert.org/fileadmin/Tmpl/documents/downloads/Remuneration_Study_2016.pdf

5 https://help.openai.com/en/articles/10032626-prompt-engineering-best-practices-for-chatgpt

6 https://help.openai.com/en/articles/6654000-best-practices-for-prompt-engineering-with-the-openai-api

7 The version of GPT-4 used is 0613: Snapshot of gpt-4 from June 13th 2023, with function calling data. 8,192 tokens. Training data up to Sep 2021.

8 EU-regulated banks must integrate the outlined procedures, such as the committee-assisted decision-making from Article 74, into their risk management.

9 IEC 61508 is a widely adopted international standard for safety-critical electronic components (Commission 2010).

10 Since the FICO HELOC Dataset does not include age as a feature, we add it.

11 In this example, the paragraph indexes are relative to the ranking of the information retriever and not the AI Act contents.
